# Reply

**DOI:** 10.1016/j.jacig.2024.100391

**Published:** 2024-12-21

**Authors:** Simon Høj, Howraman Meteran

**Affiliations:** a Department of Otorhinolaryngology, Head and Neck Surgery, and Audiology, Rigshospitalet, Copenhagen University, Copenhagen, Denmark; b Department of Public Health, Environment, Occupation, and Health, Aarhus University, Aarhus, Denmark; c Department of Respiratory Medicine, Copenhagen University Hospital-Hvidovre, Hvidovre, Denmark; d Department of Respiratory Medicine, Zealand University Hospital Roskilde-Næstved, Næstved, Denmark

To the Editor:

We appreciate the constructive feedback provided by Kleebayoon and Wiwanitkit^1^ on our recent article, “Evaluating the scientific reliability of ChatGPT as a source of information on asthma.”^2^ Their insights raise important considerations on the evaluation framework for assessing AI-driven models in health care, particularly the need for a robust and multifaceted approach.

First, we acknowledge their concerns regarding potential biases in question selection. Although thematic diversity is essential in fully evaluating any information source, the aim of our study was not to assess ChatGPT across an exhaustive set of topics but rather to examine its reliability in answering typical patient queries about asthma. These questions were carefully selected on the basis of frequency and relevance in clinical settings, ensuring that our assessment focused on information that ChatGPT is likely to provide to users. Expanding thematic scope is an interesting avenue for future research, yet for practical assessment of patient-pertinent information, we believe that our design is well justified.

Regarding the evaluation methodology, our accuracy rating scale was intentionally chosen for simplicity and ease of interpretation. Although we recognize that such a scale may not capture the full complexity of medical information, it remains a widely used metric in AI evaluation studies.^3^, ^4^, ^5^, ^6^ Future work could indeed benefit from integrating more detailed qualitative data to capture nuances in response quality. We also share the interest that Kleebayoon and Wiwanitkit^1^ have expressed in ensuring consistency across raters. We used a panel of 5 professionals with expertise in asthma care who independently evaluated responses, achieving moderate agreement as measured by the Fleiss kappa (κ = 0.42; *P* < .001). However, further standardization could enhance interrater reliability.

In response to the suggestion calling for follow-up question analysis, we emphasize that our study’s primary objective was to assess the performance of ChatGPT on first-response accuracy, as initial responses are most critical in health care information settings. Introducing follow-up interactions, although insightful, would shift the focus from the ability of ChatGPT to provide reliable primary answers to a more complex analysis of AI response progression. This approach was beyond our study’s scope, which prioritized assessing initial accuracy and the potential for patient comprehension. Nonetheless, future studies may indeed benefit from evaluating response sequences to examine depth and coherence over extended interactions.

Additionally, we support the recommendation of Kleebayoon and Wiwanitkit^1^ calling for exploring improvements in model training, such as creating a feedback loop for continuous refinement based on expert input. This approach aligns with evolving AI applications in health care, in which real-time learning and updates could strengthen the reliability of AI models as information sources. Expanding ChatGPT’s language accessibility for broader global use, as suggested, also reflects an important direction for AI development.

The potential role of AI in health care communication is promising yet complex. We appreciate the emphasis that Kleebayoon and Wiwanitkit^1^ place on exploring AI's broader role in the doctor-patient relationship and its capacity for facilitating informed decision making. Incorporating studies of patient comprehension and longitudinal assessments, as suggested, would undoubtedly enhance the rigor of future AI evaluations in health care.

We are grateful for the opportunity to respond to these valuable points raised by Kleebayoon and Wiwanitkit.^1^

## Disclosure statement

Disclosure of potential conflict of interest: H. Meteran reports receiving honoraria for lectures or advisory board meetings from GSK, Teva, Novartis, Sanofi-Aventis, Airsonett AB, and ALK-Abelló Nordic A/S within the past 5 years, unrelated to this study. H. Meteran has received research grants from ALK-Abelló A/S outside this study.
